# Persistence of SARS-CoV-2 RNA shedding and infectivity in immunized population: Prospective study along different epidemiological periods in Argentina

**DOI:** 10.1371/journal.pone.0285704

**Published:** 2023-05-17

**Authors:** Clara Theaux, Yamila Martin, Luciana Montoto Piazza, Gretel Wenk, Guillermo Notaristefano, Laura Miño, María Eugenia Sevilla, Valeria Aprea, Aldana Claps, Mercedes Nabaes Jodar, Dolores Acuña, Melina Salvatori, Laura Álvarez, María Eugenia Langan, Gabriela Turk, Ricardo Del Olmo, Mariana Viegas, Miriam Bruno, Vivian Bokser

**Affiliations:** 1 División Laboratorio, Departamento de Diagnóstico y Tratamiento, Hospital General de Agudos Dr. Carlos G. Durand, Ciudad Autónoma de Buenos Aires, Argentina; 2 Hospital Municipal de Rehabilitación Respiratoria María Ferrer, Ciudad Autónoma de Buenos Aires, Argentina; 3 Departamento de Diagnóstico y Tratamiento, Hospital General de Niños Pedro de Elizalde, Ciudad Autónoma de Buenos Aires, Argentina; 4 División Promoción y Protección de la Salud, Hospital General de Niños Pedro de Elizalde, Ciudad Autónoma de Buenos Aires, Argentina; 5 Laboratorio de Virología, Hospital de Niños Dr. Ricardo Gutiérrez, Ciudad Autónoma de Buenos Aires, Argentina; 6 Consejo Nacional de Investigaciones Científicas y Técnicas (CONICET), Ciudad Autónoma de Buenos Aires, Argentina; 7 Consorcio Argentino de Genómica de SARS-CoV-2 (Proyecto PAIS), Ministerio de Ciencia, Tecnología e Innovación, Ciudad Autónoma de Buenos Aires, Argentina; 8 Instituto de Investigaciones Biomédicas en Retrovirus y SIDA (INBIRS), CONICET–Universidad de Buenos Aires, Ciudad Autónoma de Buenos Aires, Argentina; 9 Facultad de Medicina, Universidad de Buenos Aires, Ciudad Autónoma de Buenos Aires, Argentina; 10 División Promoción y Protección de la Salud, Hospital General de Agudos Dr. Carlos G. Durand, Ciudad Autónoma de Buenos Aires, Argentina; 11 Facultad de Medicina, Departamento de Microbiología, Parasitología e Inmunología, Universidad de Buenos Aires, Ciudad Autónoma de Buenos Aires, Argentina; Centre de Recherche Scientifique et Technique sur les Regions Arides, ALGERIA

## Abstract

During the pandemic of COVID-19, numerous waves of infections affected the two hemispheres with different impacts on each country. Throughout these waves, and with the emergence of new variants, health systems and scientists have tried to provide real-time responses to the complex biology of SARS-CoV-2, dealing with different clinical presentations, biological characteristics, and clinical impact of these variants. In this context, knowing the extent period in which an infected individual releases infectious viral particles has important implications for public health. This work aimed to investigate viral RNA shedding and infectivity of SARS-CoV-2 beyond 10 days after symptom onset (SO). A prospective multicenter study was performed between July/2021 and February/2022 on 116 immunized strategic personnel with COVID-19 diagnosed by RT-qPCR, with asymptomatic (7%), mild (91%) or moderate disease (2%). At the time of diagnosis, 70% had 2 doses of vaccines, 26% had 2 plus a booster, and 4% had one dose. After day 10 from SO, sequential nasopharyngeal swabs were taken to perform RT-qPCR, viral isolation, and S gene sequencing when possible. Viral sequences were obtained in 98 samples: 43% were Delta, 16% Lambda, 15% Gamma, 25% Omicron (BA.1) and 1% Non-VOC/VOI, in accordance with the main circulating variants at each moment. SARS-CoV-2 RNA was detected 10 days post SO in 57% of the subjects. Omicron was significantly less persistent. Noteworthy, infective viruses could not be isolated in any of the samples. In conclusion, a 10-days isolation period was useful to prevent further infections, and proved valid for the variants studied. Recently, even shorter periods have been applied, as the Omicron variant is prevalent, and worldwide population is largely vaccinated. In the future, facing the possible emergence of new variants and considering immunological status, a return to 10 days may be necessary.

## Introduction

At the end of 2019, a new betacoronavirus, Severe Acute Respiratory Syndrome Coronavirus 2 (SARS-CoV-2), emerged in the human population with unusual characteristics regarding its rapid global spread, high transmissibility, requirement for hospitalizations and number of deaths, thus causing the coronavirus infection disease (COVID-19) to become pandemic [1].

During the two and a half years of the pandemic of COVID-19 (2020–2022), numerous waves of infections affected the two hemispheres with different impacts on each country. During the first stages of the pandemic, health systems collapsed throughout the world, leading governments and Public Health agencies in each country to take drastic containment measures that, in many cases, led to strict confinement. Argentina reported the first cases of COVID-19 in early March 2020 and experienced an initial wave which lasted from May 2020 to February 2021, in a context of a severe lockdown with restrictions on internal movement during most of that period [2]. The molecular epidemiology of the circulating SARS-CoV-2 strains during this wave was characterized by many lineages highly similar to the index virus (WIV04, GISAID accession number EPI_ISL_402124). There was a significant second wave from March to October 2021, driven throughout the country mainly by the Gamma and Lambda variants, although it showed a heterogeneous distribution. Towards the end of this second wave, the Delta variant was introduced and displaced the others. Subsequently, there was a third wave (November 2021-March 2022) initially driven by the Delta variant; however, the emergence of the Omicron variant, with a greater transmissibility, resulted in the total displacement of Delta between epidemiological weeks (EW) 1–3 of 2022 in all the provinces of the country [3, 4].

Throughout the different waves and stages of the pandemic, and with the emergence of new variants, health systems and scientists have tried to provide real-time responses to the complex biology of SARS-CoV-2, dealing with different clinical presentations, biological characteristics, and clinical impact of these variants. This led to constant changes in clinical case definitions, diagnostic protocols, isolation period of positive cases and close contacts, etc. In this context, knowing the extent period in which an infected individual releases infectious viral particles has important implications for public health [5, 6].

Public health policies implemented in Argentina had been very dynamic, trying to optimize human and material resources. One of the questions raised was the isolation period required for health workers and strategic personnel testing positive for SARS-CoV-2, as they were essential to face the pandemic. Since August 2020, the isolation period indicated for a subject with a positive RT-qPCR test was 10 days from the onset of symptoms, being clinically discharged without the need of a negative molecular test. That resolution remained valid in the context of Gamma and Lambda as main circulating variants in May 2021 [3, 7]. Towards the end of the third wave, this period was shortened to 5 days of home isolation plus 5 days of extreme care at the workplace [8]. This occurred in the context of massive vaccination, which started in December 2020 with health and strategic personnel as the first target population to be vaccinated [9].

Today, it is widely known that detection of SARS-CoV-2 RNA and even shedding of infectious viral particles can persist beyond symptom resolution. Different authors described persistently positive SARS-CoV-2 RT-qPCRs for weeks or months after clinical recovery (a usual finding in clinical practice), that generally do not reflect the presence of viruses with infectious capacity [10–12]. Although some reports are contradictory, the period of persistence seems to depend on disease severity, age, immune status, and other factors [12–16]. However, further research is needed to understand the duration of viral RNA shedding and potential infectivity among vaccinated individuals (especially for those with mild to moderate COVID-19), and to determine if these findings can be extended to all VOC and VOIs.

The aim of this work was to determine the association of SARS-CoV-2 RNA persistence in nasopharyngeal swabs and the shedding of infectious viral particles in healthcare workers and other strategic personnel diagnosed as asymptomatic, mild or moderate COVID-19, after 10 days from the symptoms onset date. The strength of this study relies on the evaluation of these parameters along different epidemiological periods of the pandemic, where different viral variants circulated in the Metropolitan Area of Buenos Aires (Buenos Aires and surroundings), Argentina.

## Materials and methods

### Study design, target population and ethical statement

This was a multicentric prospective descriptive longitudinal observational study aimed at evaluating SARS-CoV-2 RNA and viral shedding persistence in respiratory samples, performed between July 2021 and February 2022. The project was approved by the different ethic committees corresponding to all sites of enrollment (PRIISA codes 5162, 5163, 5220). All participants signed an informed consent.

#### Inclusion criteria

The target population was defined as immunized adults (>18 years old) diagnosed with SARS-CoV-2 infection by RT-qPCR, including healthcare workers and other essential workers who attended Febrile Urgency Units (FUUs) or emergency departments at the Pedro de Elizalde Children’s General Hospital, María Ferrer Respiratory Rehabilitation Hospital and Dr. Carlos G. Durand Acute General Hospital in Buenos Aires City, Argentina. Healthcare and essential workers were selected as the target population in order to gain adherence to the project, and also since they were the first group eligible for vaccination in Argentina.

Healthcare workers were defined as any person who worked in public or private health care institutions, including physicians, nurses, administrative, logistics and cleaning assistants, ambulance drivers and every healthcare worker with or without an academic degree. Other essential workers belonged to the educational system and security forces.

#### Other inclusion criteria

Both symptomatic and asymptomatic individuals with at least one dose of SARS-CoV-2 vaccine applied at least 21 days before the onset of symptoms or before the date of diagnosis (in asymptomatic patients); and with Ct values <30 when diagnosed by RT-qPCR.

#### Exclusion criteria

Subjects with positive SARS-CoV-2 RT-qPCR in the previous 45 days; pregnant and immunocompromised individuals on any cause (oncological diseases, chemotherapy, HIV, high dose corticoid administration or others).

Nasopharyngeal Swab 1 (NPS-1) was the SARS-CoV-2 diagnostic sample, which was also used for sequencing to identify SARS-CoV-2 genetic variant. Day 0 was defined as the symptom onset date (or the date of first positive sample for asymptomatic subjects). Potential participants were contacted by phone to attend the hospital’s FUUs to sign informed consent and to take a second NPS (NPS-2) after 10 to 14 days. NPS-2 samples with RT-qPCR Cts<35 were analyzed for viral infectivity; those with RT-qPCR Cts results below 30 were attempted to be re-sequenced, in order to identify SARS-CoV-2 genetic variants or mutations acquired in the Spike protein in comparison with the first sequenced sample. When NPS-2 resulted in Cts over 35 or non-detectable, follow-up was ended. When NPS-2 resulted with a Cts<35, the subjects were asked to attend the FUUs for a third NPS (NPS-3) after 15 to 17 days (minimum 5 days after NPS-2). NPS-3 results were processed in the same manner as previous NPS. All subjects with NPS-3 resulting in Cts<35 were asked to attend the FUUs for a fourth NPS (NPS-4) after 20 to 24 days (minimum 5 days after NPS-3). NPS-4 results were the last to be considered for the analyses.

### SARS-CoV-2 detection in clinical samples

The clinical samples obtained from enrolled participants were nasopharyngeal swabs (NPS) collected in 1 ml of sterile physiological solution and were sent to the Elizalde and Durand Hospitals’ molecular biology laboratories for diagnosis.

Viral RNA was extracted with automated systems and RT-qPCR was performed with the DisCoVery SARS-CoV-2 RT-PCR detection kit Cy5 (Transgen Biotech) following manufacturer’s instructions.

All SARS-CoV-2 RT-qPCR results were validated by RNAse P as a sample’s cellularity constitutive control, multiplexed with the specific SARS-CoV-2 target genes. RNase P Cts obtained were all between 29 and 32, therefore all samples were considered to have equivalent and acceptable quality.

### SARS-CoV-2 positive samples sequencing

NPS-1, NPS-2, and NPS-3 with RT-qPCR Ct<30 were partially sequenced for protein Spike (S) coding gene through Sanger traditional method, using CDC’s recommended sequencing protocol, amplifying segment 29 (fragment comprised between amino acids S_248 and S_750) [17]. This region allows the identification of signature mutations associated with variants Alpha, Beta, Gamma, Delta, Lambda, and Omicron and Non-VOC/VOI.

### SARS-CoV-2 isolation in cell cultures

The presence of infectious viral particles in NPS-2, 3 and 4 with Ct<35 was evaluated by inoculating Vero E6 cell cultures. After the initial inoculation, cell culture supernatants were harvested and inoculated into freshly prepared cells every 3 days. The presence of cytopathic effect (CPE) was monitored at each passage. At the third passage (or upon the appearance of CPE), cell culture supernatants were collected and SARS-CoV-2 RT-qPCR were performed in order to confirm or exclude viral isolation. All work involving infectious SARS-CoV-2 was conducted at the BSL3 facility located within the INBIRS institute. Upon sampling, NPS dedicated to virus isolation were immediately frozen at -80°C and transported in dry ice to the INBIRS institute when appropriate. Within one or two days, samples were thawed for the first time, filtered and seeded into cells. Sample handling, cell culture preparation and inoculation, CPE visualization and supernatant harvesting were performed by trained and expert personnel, following the institutional biosafety guidelines and regulations.

### Epidemiological data

All demographic and vaccination data were obtained from the Buenos Aires City Government’s electronic medical history system (SIGEHOS) and the national sanitary data system *Sistema Integrado de Información Sanitaria Argentina* (SISA). Clinical data was obtained from direct individual interviews and SIGEHOS.

### Statistical analysis

Statistical analysis was performed with STATA 8.0 version [18].

Comparison of two samples of categorical variables was performed by Chi square; comparison of two samples of continuous data by two tailed t-tests or Wilcoxon rank sum tests.

Data distribution normality was evaluated by Shapiro-Wilk test (skewness, kurtosis and histogram).

Comparison of two or more groups of numeric independent variables was performed by ANOVA or Krustal Wallis. Homoscedasticity was checked by Barlett and Levene tests. Bonferroni method was used for corrections. P-values lower than 0.05 were considered significant.

### Sequencing data availability

The partial spike protein gene sequences generated in this study were submitted to EpiCoV (GISAID) (ID: EPI_ISL_5656726- EPI_ISL_5656734; EPI_ISL_5656770; EPI_ISL_5656778- EPI_ISL_5656780; EPI_ISL_5656782- EPI_ISL_5656784; EPI_ISL_5656870; EPI_ISL_5656929; EPI_ISL_5657062; EPI_ISL_5657063; EPI_ISL_5657104; EPI_ISL_5657105; EPI_ISL_5657190; EPI_ISL_5657194; EPI_ISL_5657257- EPI_ISL_5657259; EPI_ISL_5657307; EPI_ISL_5657308; EPI_ISL_5657354; EPI_ISL_5657396; EPI_ISL_5657735; EPI_ISL_5657933; EPI_ISL_5657940; EPI_ISL_5657943; EPI_ISL_5658030; EPI_ISL_16238167- EPI_ISL_16238228; EPI_ISL_16239395).

## Results

### Study group description

A total of 116 subjects were included in the study between July 2021 and February 2022. The median age was 39 years and 67% were females. Ninety-three subjects (80%) were health workers, 15 (13%) educational workers, and 7 (7%) security force workers. Of the 116 subjects, 56% lived in Buenos Aires City, and 44% in the Metropolitan Area of Buenos Aires.

Regarding the clinical features of the subjects, 91.5% had a mild disease, 2.5% a moderate disease and 7% were asymptomatic. At the time of COVID-19 diagnosis, 74.1% of the subjects had two vaccine doses, 21.6% had two doses plus a booster dose and 4.3% had only one dose. Table 1 also describes the vaccine type or combination administered, with two Sputnik doses being the most prevalent scheme (50% of subjects), followed by two Sputnik and one AstraZeneca booster (16.4%). Median of days between the last vaccine dose and diagnosis date was 130, with a minimum of 9 and a maximum of 365 days.

**Table 1:** Vaccination scheme and types of vaccines administered at the time of COVID-19 diagnosis in enrolled subjects.

Number of Doses (Vaccine type and tradename)	Number of cases, n (%)
1 Dose	5 (4.3%)
GAM-COVID-Vac (Sputnik V)	2 (1.7%)
ChAdOx1 nCoV-19 (AZD1222) (Oxford—AstraZeneca)	3 (2.6%)
2 Doses	86 (74.1%)
GAM-COVID-Vac (Sputnik V)	58 (50%)
ChAdOx1 nCoV-19 (AZD1222) (Oxford—AstraZeneca)	15 (12.9%)
BBIBP-CorV (Sinopharm)	13 (11.2%)
3 Doses	25 (21.6%)
2 GAM-COVID-Vac (Sputnik V) + 1 ChAdOx1 nCoV-19 (AZD1222) (Oxford—AstraZeneca)	19 (16.4%)
2 GAM-COVID-Vac (Sputnik V) + 1BNT162b2 (Pfizer-BioNTech)	1 (0.9%)
2 GAM-COVID-Vac (Sputnik V) + 1 mRAN-1273 COVID-19 (Moderna)	1 (0.9%)
2 BBIBP-CorV (Sinopharm) + 1 ChAdOx1 nCoV-19 (AZD1222) (Oxford—AstraZeneca)	4 (3.4%)
Total of subjects	116 (100%)

### SARS-CoV-2 RNA persistence beyond 10 days post-symptoms onset in vaccinated subjects

With the aim of identifying the frequency of detectable SARS-CoV-2 RNA in NPS beyond the recommended isolation period valid at the time that this study was performed, follow-up samples were obtained in enrolled participants and were subjected to the RT-qPCR.

**Fig 1:**
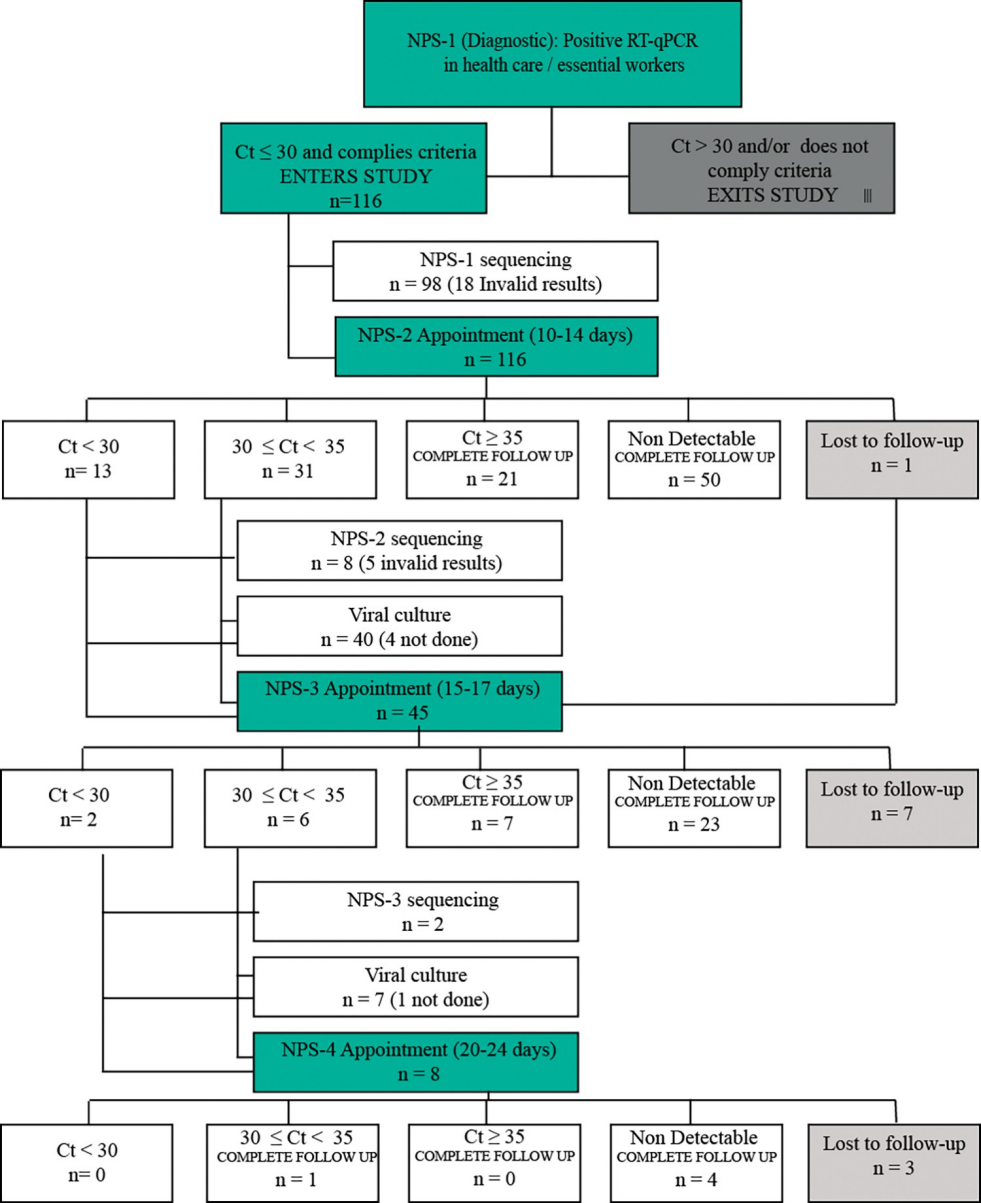
General procedure and results of the study.

Fig 1 shows the general procedure and results of the study over time according to the number of NPS taken, RT-qPCR results classified by N gene Ct values, number of sequenced samples, and samples subjected to viral culture. The number of subjects who were lost to follow-up is also shown.

Regarding the procedure, NPS-1 were taken with a median of 1 day post symptom onset (or post diagnosis date for asymptomatic subjects) (IQR = 1–2); the mean of N gene Ct values was 21.05 (SD 4.2), in a range from 12.1 to 31.

NPS-2 were taken with a median of 11 days post symptoms onset (IQR = 11–13). Out of the original 116 samples, 50 NPS-2 (43%) resulted negative by RT-qPCR, 66 (57%) resulted positive, and there was 1 lost subject who later continued for NPS-3. N gene Ct values of positive samples had a mean of 32.64 (SD 3.4) in a range of 21 to 38.

A total of 45 subjects were appointed for NPS-3 between days 15 to 17. Seven subjects were lost to follow-up at this stage (15.5%), and 38 samples were taken with a median of 16 days post symptom onset (IQR = 15–17). Twenty-three of these (60.5%) resulted negative and 15 (39.5%) resulted positive by RT-qPCR. N gene Ct values had a mean of 33.5 (SD 4.6) in a range of 21.5 to 40.

NPS-4 was indicated for 8 subjects between days 20 to 24. Three subjects were lost to follow-up and 5 samples were taken with a median of 20 days post symptoms onset (IR = 20–20). Four of these resulted negative and 1 resulted positive with an N gene Ct = 33.6.

**Fig 2:**
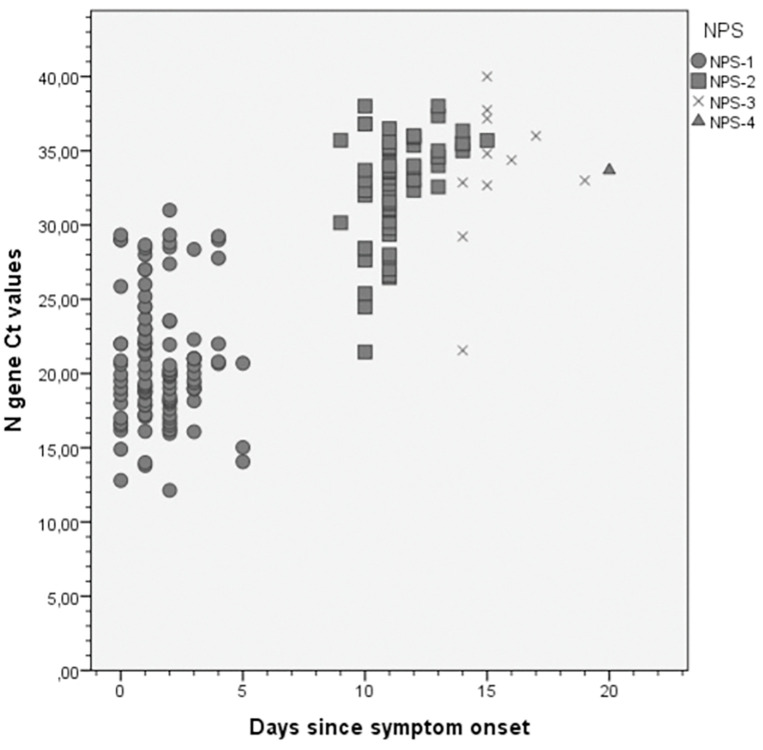
N gene Ct values vs symptom onset date in positive RT-qPCR samples.

In summary, Ct mean values slightly arose over time, but Ct ranges were overlapped for NPS-2, 3 and 4. No statistically significant differences were found between NPS-2 and NPS-3 mean N gene Cts, by t test (p = 0.5). Fig 2 depicts N gene Ct values over time for all positive samples, reinforcing that Ct values are highly variable on the same days since symptom onset, at least between NPS-2, 3 and 4.

Nevertheless, individual analysis of samples of the same subject showed that Ct values of N gene increased between NPS-1 to 4, reflecting a lower amount of viral RNA in the nasopharynx over time. Also, 3 samples kept Ct values below 25 even after 10 days of infection.

### SARS-CoV-2 sequencing at enrollment and in samples with persistently detectable viral RNA beyond 10 days post-symptoms onset

Then, we aimed to determine the SARS-CoV-2 viral variants in the samples at the time of enrollment and in persistently detectable follow-up samples (Ct<30) to detect variant changes or mutations acquired in the Spike protein in comparison with the first sequenced sample.

By the end of the study, 98 NPS-1 samples were successfully sequenced: 15 were Gamma (15.3%), 16 Lambda (16.3%), 42 Delta (42.9%) and 24 Omicron (24.5%) (Table 2). Within Omicron variant, Sanger sequencing allowed us to determine that all the sequences belonged to BA.1 lineage.

**Table 2:** SARS-CoV-2 variant distribution according to NPS in positive samples.

	NPS-1 (Diagnostic sample)		NPS-2 (Days 11 to 15)		NPS-3 (Days 16 to 19)	
**Variant (lineage)**	**Number of cases, n**	**% of total sequenced samples**	**Number of cases, n**	**% of total sequenced samples**	**Number of cases, n**	**% of total sequenced samples**
**Gamma(P1)**	15	15.3	1	12.5	0	0
**Lambda(C.37)**	16	16.3	1	12.5	1	50
**Delta(B.1.617.2)**	42	42.9	4	50	1	50
**Omicron(BA.1)**	24	24.5	2	25	0	0
**Non-VOC/VOI**	1	1.0	0	0	0	0
**Total**	98	100	8	100	2	100

Eighteen NPS-1 and 5 NPS-2 samples did not provide results, 9 of which had RT-qPCR Cts~28 suggesting the need of a higher viral RNA amount for successful sequencing.

As shown in Table 2, 8 NPS-2 resulted in successful sequencing. In addition, 2 of these 8 samples were successfully sequenced in NPS-3. Neither changes of SARS-CoV-2 variant were found within the same COVID-19 event in any of the enrolled subjects, nor were additional amino acids changes in the sequenced region of the spike protein gene detected.

Table 3 shows Ct mean values in relation to variants for NPS-1 (diagnostic sample) and NPS-2. There were no statistically significant differences in Ct mean values among the different variants studied. In order to understand if viral variants were associated with different persisting rates, we analyzed the proportions of persistently positive NPS-2 according to viral variants in patients with 2 vaccine doses and no previous COVID-19 event: 40% of Delta (17/42), 36% of Gamma (5/14), 46% of Lambda (6/13) and 0% of Omicron samples (0/7) presented RT-qPCR persistently positive in NPS-2. When comparing Omicron persistence versus the rest of the variants by the Chi square Test, Omicron was significantly less persistent (p = 0.03).

**Table 3:** Ct values in relation to variants for NPS-1 and NPS-2.

NPS	Variant (n)	N gene Ct Values Mean (SD)	Statistical Significance^1^
**NPS-1**	Gamma (n = 13)	20.3 (3.6)	P = 0.202 NS
	Lambda (n = 13)	21 (3.5)	
	Delta (n = 39)	19.4 (3.8)	
	Omicron (n = 5)	20.5 (2.5)	
**NPS-2**	Gamma (n = 8)	32.2 (3.6)	P = 0.667 NS
	Lambda (n = 8)	33.4 (3)	
	Delta (n = 23)	32 (3.6)	
	Omicron (n = 2)	36.5 (2)	

^1^Analysis of Variance (ANOVA), NS = non-significant

Throughout the entire sampling period, 3 different epidemiological moments can be described. Thus, participants were classified according to the period in which they were enrolled for certain analysis: period 1 from July to September 2021, with the predominant variants Gamma and Lambda (32% of the subjects); period 2 from October to December 2021, with Delta variant as the most prevalent (42% of the subjects); and period 3 from January to February 2022, with Omicron variant as the only one detected (24%). One subject was infected with a Non-VOC/VOI variant in August 2021. No differences were found in the percentages of sequenced variants in relation to those that were mostly circulating in the community at that time, regardless of the type of vaccine and vaccination status.

### Viral infectivity in samples with persistently detectable RNA beyond 10 days post-symptoms onset

The shedding of infectious viral particles in samples with persistent RNA detection was studied by inoculating the NPS-2, 3 and 4 (with Ct<35) into cell cultures. Forty NPS-2s and 7 NPS-3s were tested. None of these cultures developed CPE or had detectable SARS-CoV-2 RNA, failing to detect infectious SARS-CoV-2 particles in any of the culture supernatants. Although one NPS-4 met the criteria for viral culture (detectable RNA with a Ct<35), this sample was not cultured since previous samples of the same subject were consistently negative for CPE in NPS-2 and NPS-3.

## Discussion

In this study, we performed an active epidemiological study of SARS-CoV-2 RNA persistence and infectivity in NPS samples of healthcare and other essential workers who received at least one dose of vaccine during a period of 9 months (July 2021-February 2022) which encompassed the sequential circulation of Gamma, Lambda, Delta, and Omicron variants.

Regarding the SARS-CoV-2 RNA persistence, we found no statistically significant differences between Cts mean values in the different NPS over time. Even though we applied the same procedure and used the same RT-qPCR kit in all the samples throughout the study, nasopharyngeal swabs have an intrinsic variability that depends on the operator and the tolerance of the patients to be swabbed. In any case, all the assays in this study were validated by RNAse P as a cellularity constitutive control of the samples. In addition, although the analysis of each individual case showed that Ct values were higher as days passed, reflecting a lower amount of viral RNA in nasopharynx while clinical symptoms improved, some participants kept Ct values below 25 even after 10 days. Taking into account that RNA has been persistently detected by RT-qPCR after acute infections in different viral infections and that neither the integrity of the genome nor the infectivity of the viral particle are guaranteed, we consider that Ct values should not be used as the only parameter to establish active infection, transmission potential, nor disease progression. Nevertheless, it has been shown that many respiratory viruses present mechanisms that allow viral spread within the airway epithelia without particle release, as an alternative mechanism of spread [19]. In this regard, there is still much to know about viral and host determinants of infectivity and pathogenesis [20].

When discussing viral persistence, there are two scenarios: viral RNA detection in respiratory secretion samples and in deep tissues. In patients with severe infections, viral RNA detection in these two sample locations may not necessarily concur. Post-mortem analysis in patients who experienced persistent severe respiratory symptoms has shown viral RNA detection in peribronchial glands and bronchial cartilage infected cells, even up to 300 days after the initial episode, despite several negative PCR results in nasopharyngeal and bronchoalveolar lavage samples [21]. It is important to note that such patients were not included in our study and that we are referring to persistent viral shedding based on genetic material detection in upper respiratory samples.

Despite its limitations, viral isolation in cell culture is still the gold standard technique to detect infectious particles. Thus, we attempted to isolate infectious SARS-CoV-2 through cell culture technique from 47 NPS obtained as part of this study. No cytopathic effect was observed in any of the cultures inoculated with NPS taken after day 10, even in those with low Cts (minimal Ct was 21). Unsuccessful viral isolation due to unsuitable storage conditions, improper handling or other methodological issues is highly unlikely based on the training and documented expertise of the personnel in charge of these experiments. On the contrary, these findings reinforce the notion that the detection of SARS-CoV-2 RNA is not necessarily related to the presence of replication competent viral particles, at least after 10 days. These results apply to immunocompetent individuals with mild or moderate disease who received at least 1 vaccine dose and can be extended to the different VOC and VOIs evaluated here. It should not be extended to persons with severe COVID-19, elderly [22] or immunocompromised people. Nevertheless, that is an interesting observation as it reinforces the use of evolution days since symptoms onset date for estimating the infectious period, instead of a negative RT-qPCR result as it was done in the first stages of the pandemic. Noteworthy, different studies support these findings. Failure to detect replication-competent virus ≥10 days after symptom onset in individuals with mild to moderate COVID-19 has been previously reported in unvaccinated individuals [23]. In addition, another report indicated that the probability of viral culture positivity was 18% between days 7 and 9, and 0% from day 11 [24], and a study performed in Taiwan concerning Omicron variant’s secondary attack rate, demonstrated that 99,1% of subjects had been in contact with the index case before 10 days since symptoms onset date [25]. More recently, no viral isolates were obtained 10 days after diagnosis in vaccinated people infected with the Delta and Omicron variants [26, 27].

However, it should be acknowledged that viral culture has low sensitivity. Thus, a negative isolation does not completely exclude the presence of replication-competent viruses. Detection of infectious virus is inherently less sensitive than detection of viral RNA and may be influenced by the presence of neutralizing antibodies in the sample, especially in vaccinated individuals such as the target population of this study. It has been reported (even for other respiratory viruses) that qPCR can detect genomic fragments that could correspond to viral particles without replication capacity, or genomic debris. However, successful sequencing in samples after 10 days implies the presence of long genome fragments that may not be genomic debris, as it was found in this study. An alternative to viral culture would be to perform more sensitive techniques that can easily detect active viral replication, as the detection of subgenomic RNA (sgRNA) [28], proposed as active replication markers since they are not packaged into virions [29]. A study carried out in Hong Kong reveals that SARS-CoV-2 infectious particles and sgRNA were rarely detectable beyond 8 days after onset of illness, although viral RNA was detectable for many weeks by RT-qPCR [30]. In other studies, sgRNA was detected in diagnostic samples up to 17 days after initial detection of infection [31], probably owing to the stability and nuclease resistance of double-membrane vesicles containing these sgRNAs. Therefore, although the absence of sgRNA would indicate the absence of viral replication, the presence of sgRNA does not necessarily indicate infectiousness [32]. Thus, although this type of molecular analysis would be able to provide supplementary data regarding the differential persistence of different viral RNA forms and its association with viral infectivity, it cannot replace viral isolation in cell culture as the gold-standard technique to assess infectivity.

A cohort study in mild and moderate patients that included different variants, comparatively evaluated viral persistence through culture, sgRNA, and genomic RNA [33]. In this study, cultures became negative early around day 6, followed by sgRNA at day 11 and genomic RNA at day 18. Although SARS-CoV-2 sgRNAs in nasopharyngeal swab samples could be detected after the period of isolation of viable virus, sgRNAs declined faster than genomic RNAs. When analyzing factors associated with increased persistence of genomic RNA, two vaccine doses had a protective effect on genomic RNA persistence, while steroid use was associated with prolonged viral shedding measured through culture and sgRNA [33]. Consistent with these findings, in our study with vaccinated population, 43% of samples analyzed in the second swab were non-detectable, increasing to 85% in NPS-3 around day 16.

As determined by viral sequencing, the most prevalent variant detected in our study was Delta, followed by Omicron, Gamma and Lambda. Each variant was prevalent in the period of highest circulation in Buenos Aires city and surroundings, according to data from Argentina [3, 4, 34].

Our study population, composed of immunocompetent individuals, did not reveal the presence of escape mutations in subsequent samples sequenced over time. This can be attributed to the relatively low mutation rate of the virus, which, combined with the short time interval between samples, did not allow enough time for mutations to occur and become fixed within the context of global transmission in an immunocompetent population [35]. In contrast, other studies performing quasi-species analysis, even before the emergence of VOCs, have shown high intra-patient variability in severe and immunosuppressed patients with persistent or prolonged viral shedding [36].

On the one hand, there was no statistically significant difference in Ct mean values between different variants in NPS-1 and NPS-2 in positive samples, as shown by other studies [37, 38]. On the other hand, analyzing positivity in subsequent samples for different variants, Omicron (BA.1) was significantly less persistent than the other ones [39]. This raises some questions regarding the reasons behind Omicron’s noticeable higher transmissibility worldwide. It has been reported that Omicron BA.1 caused shorter viral RNA shedding and lower peak viral RNA concentrations in comparison to Delta variant [40, 41], as well as shorter symptomatic infections [39]. In contrast, other studies found a similar RNA viral load for Omicron-infected and Delta-infected patients [42, 43]. These results suggest that the observed higher transmissibility of Omicron BA.1 is unrelated to an increased shedding of infectious viral particles, in agreement to our own results [36].

Another group studied the replication competence and cellular tropism of SARS-CoV-2 variants in ex vivo explant cultures of human bronchi, and reported a faster and enhanced viral replication efficiency for Omicron compared to the previous lineages [44]. In a recently published article, the authors showed that Omicron variant (BA.1) enters and replicates more efficiently in the nasal epithelium than D614G and Delta variants, possibly due to an enhanced interaction with ACE2 receptors and the ability to spread within the nasal epithelium without a lag phase [45]. All this data suggest that Omicron has an intrinsic biological capacity for enhanced transmission.

Due to the dates of recruitment, most of the participants had 2 vaccine doses at the time of infection. The circulation periods for each variant and the vaccine availability in Argentina should be considered: Gamma and Lambda variants were predominant during the first stage of the second wave of COVID-19 in Argentina (March 2021 –July 2021), when health care workers in Argentina had already been primarily vaccinated with the first 2 doses of Sputnik vaccine (the first one available in Argentina). Infections with Gamma, Delta and Lambda variants were only found in participants with 2 vaccine doses, since booster doses were still not available in Argentina in the period of co-circulation of these variants (late August 2021-October 2021). Omicron variant was predominant in late December 2021—January 2022, when the third dose (or booster dose), predominantly AstraZeneca, was available for those with a second dose applied more than 4 months before. A study conducted in the United States showed that breakthrough infections with Omicron variant were more likely, even in subjects with a booster dose, probably due to immune escape [46]. Overall, variants found in our study were coincident to the most frequently circulating variants in the community at each time, regardless of the type of vaccine and vaccination status [4, 34].

One of the limitations of this study is that it was not possible to determine if the infectious period could be even shorter, since the isolation period established at the beginning of this study was 10 days, therefore participants could not attend to the FUUs for shorter sampling periods. While this study was nearly coming to its end, the Public Health Ministry of Buenos Aires City reduced the isolation period from 10 to 5 days, adding 5 days of extreme care to prevent transmission.

In summary, SARS-CoV-2 RNA persistence can be observed even after 10 days post-symptoms onset in immunized individuals infected with SARS-CoV-2 in the context of VOC and VOI variants circulation (Gamma, Lambda, Delta and Omicron), but this persistence was not associated with the presence of replication-competent viral particles. Also, Omicron BA.1 RNA was significantly less persistent than the other variants.

## Conclusions

In conclusion, suggesting 10 days of isolation was useful to prevent further infections, and proved valid in the context of the different variants evaluated in this study. This could nowadays be considered conservative, as shorter isolation or extreme care periods have been applied in the last months, as the Omicron variant is prevalent, and worldwide population is largely vaccinated. In the future, facing the possible emergence of new variants and considering the variable immunological status of the world population (as booster applications are not always available and/or accepted), it might be necessary to return to those safe 10 days of isolation until further information is recovered.
